# Sweet Success: Using Candy Land to Explore Gamification in Medical Education

**DOI:** 10.5070/M5.53816

**Published:** 2026-10-31

**Authors:** Jessica Rachel Baez, Sierra Hajdu

**Affiliations:** * University of Cincinnati Medical Center, Department of Emergency Medicine, Cincinnati, OH

## Abstract

**Audience:**

This session is intended for learners and educators interested in medical education, including medical students, residents, fellows, and faculty.

**Introduction:**

Gamification is increasingly recognized as an effective strategy to enhance engagement, motivation, and active learning in medical education. Despite its potential, many educators are unsure how to implement gamified strategies effectively. This session demonstrates a practical, interactive approach to applying gamification principles in a collaborative educational setting.

**Educational Objectives:**

By the end of this session, learners will be able to (1) describe key principles of gamification in medical education, (2) identify resources and tools for implementing gamification, (3) actively participate in a gamified educational activity, and (4) design a simple plan to implement gamification in their own teaching, considering benefits and potential challenges.

**Educational Methods:**

The session started with brief didactic lecture on game-based learning and gamification intended to orient participants to the material. Participants then engaged in a Candy Land-inspired, team-based activity. Twenty-five prompts covering aspects of gamification were arranged along a winding pathway drawn with chalk. Teams of 3–4 learners advanced along the path by rolling a large inflatable die and answering questions or discussing scenarios associated with the prompts. Correct answers earned points or allowed teams to advance, while debriefing discussions linked gameplay to the principles and strategies of gamification. Incorrect answers were not penalized. This approach leveraged experiential learning, active learning, and social constructivist principles to support both knowledge acquisition and practical application.

**Research Methods:**

The session was evaluated through direct observation and informal feedback from learners and instructors. Engagement, discussion quality, and participation were used as primary indicators of effectiveness. Modifications and recommendations for future implementation were derived from observed challenges and learner feedback.

**Results:**

Fifteen learners of varied training levels participated. Observations indicated high engagement, lively discussion, and collaborative problem-solving. Feedback was universally positive, with learners reporting increased confidence in understanding gamification principles and motivation to apply these strategies in their own teaching. Several participants subsequently incorporated gamified activities into educational sessions in the following weeks. Key lessons for implementation included ensuring sufficient space, clarifying scoring systems, and preparing materials in advance.

**Discussion:**

This session demonstrates that gamification can effectively engage learners across multiple levels, promote collaborative learning, and provide practical insights into implementation strategies. Learners experienced the mechanics and benefits of gamification firsthand, while also reflecting on challenges and planning for real-world application. The activity highlights how structured games can create a safe and inclusive learning environment, fostering participation from all learners. Future iterations may further optimize space, scoring, and team organization to enhance learning outcomes.

**Topics:**

Gamification in medical education, game-based learning, interactive learning strategies, experiential learning, social constructivism, educational game design, team-based learning, implementation strategies, engagement and motivation, active learning.

## USER GUIDE

**Table:** 

List of Resources:	
Abstract	72
User Guide	74
Learner Responsible Content	77
Small Groups Learning Materials	78

**Learner Audience:**

learners and educators interested in medical education, including medical students, residents, fellows, and faculty

**Time Required for Implementation:**

1–2 hours

**Recommended Number of Learners Per Instructor:**

15–20

**Topics:**

Gamification in medical education, game-based learning, interactive learning strategies, experiential learning, social constructivism, educational game design, team-based learning, implementation strategies, engagement and motivation, active learning.

**Objectives:**

By the end of this 1–2-hour session, learners will be able to:

1. Describe at least three key principles of gamification in medical education.
2. Identify at least two resources (articles, websites, or tools) that can be used to gamify medical teaching.
3. Actively participate in a Candy Land-based activity to apply these principles.
  Additionally, learners will be able to design a simple plan for implementing gamification in an educational setting, considering both potential benefits and challenges. Learners will complete all activities and reflections within the session timeframe, ensuring that knowledge, application, and implementation skills are reinforced in a collaborative, interactive environment.

### Linked objectives and methods

The goals of this session were achieved through initial delivery of an introductory didactic lecture on game-based learning, and an interactive, team-based gamification activity designed to allow learners to actively apply knowledge, explore implementation strategies, and reflect on the benefits and challenges of gamification in medical education. Gamification has been shown to enhance engagement, motivation, and knowledge retention in graduate medical education.^1^,^2^,^3^

Twenty-five prompts covering various aspects of gamification were printed and affixed to construction paper, then arranged along a large, winding pathway drawn with chalk. Each prompt required learners to engage with content by answering questions, discussing scenarios, or critically thinking about gamification strategies. Prompts were organized into different themes including recall/knowledge, creativity, collaboration, energy boosters, and motivation. Each theme was paired with a specific Candy Land-inspired candy icon and corresponding construction paper color to visually reinforce the category of questions and help learners quickly recognize prompt types during gameplay. Participants were divided into small teams of 3–4 learners and took turns rolling a large inflatable die to advance along the pathway. When a team landed on a prompt, they revealed the question and attempted to answer it. Correct answers earned points or allowed teams to advance further, reinforcing both knowledge and practical application. The team that reached the final space on the pathway first earned bonus points. The team with the most points at the end of the session was recognized. Throughout gameplay, facilitators incorporated brief discussions after selected prompts to explicitly connect learner experiences back to principles introduced during the didactic lecture. For example, facilitators highlighted how specific prompts demonstrated concepts such as serious games, simulation, team-based learning, rapid feedback, or motivational game mechanics. These structured reflections were intended to help learners recognize the educational purpose behind individual game elements and reinforce how gamification strategies can be intentionally aligned with learning objectives. Facilitators were encouraged to adapt the depth and frequency of debriefing discussions based on learner engagement, available time, and participant familiarity with gamification concepts.

This format supported achievement of the session objectives by providing a highly interactive and collaborative learning environment where learners could discuss and debate gamification strategies, experience firsthand the mechanics of a gamified educational activity, and reflect on how similar approaches could be implemented in their own teaching.^4^,^5^,^6^,^7^ Learners received education on gamification design principles such as the value of integrating game-based learning with training goals, and the importance of fair game rules and rapid feedback, along with the practical application of game mechanics like points and leaderboards.^4^ The combination of team-based learning, movement, competition, and structured prompts allowed learners to connect theory with practice, reinforcing knowledge acquisition, application, and implementation planning in an engaging and memorable way.^3^,^8^,^9^

Importantly, learners were also introduced to strategies for balancing educational content with gamification elements to ensure that gameplay enhanced rather than distracted from learning objectives. Facilitators emphasized that successful gamification should prioritize alignment between educational goals, learner needs, and game mechanics. Discussion included the importance of avoiding excessive competition or overly complex rules that may detract from content delivery, while ensuring that game elements such as points, movement, and challenges directly reinforce desired learning outcomes. Learners were encouraged to view gamification as a tool to support educational objectives rather than as the primary focus of the session itself.

Conceptually, this session was designed using experiential learning and social constructivist principles to maximize engagement and knowledge retention.^7^,^9^ Learners actively applied gamification concepts rather than passively receiving information, consistent with active learning theory. The activity allowed learners to construct knowledge collaboratively, discussing strategies and reflecting on both the mechanics and educational impact of gamification, aligning with social constructivist theory.^1^,^5^ The game format also follows Kolb’s experiential learning cycle, providing concrete experience through play, reflective observation through team discussion, abstract conceptualization by linking the activity to educational principles, and active experimentation by planning how gamification could be implemented in learners’ own teaching contexts.^8^ This framework ensured that learners not only understood gamification theoretically but also gained practical insights into applying these strategies effectively in diverse medical education settings.^2^,^7^

### Recommended pre-reading for facilitator

- Watsjold BK, Cosimini M, Mui P, Chan TM. Much ado about gaming: An educator’s guide to serious games and gamification in medical education. *AEM Educ Train*. 2022 Aug 23;6(4):e10794. PMID: 36034886. PMCID: PMC9399447. doi:10.1002/aet2.10794

### Learner responsible content (LRC)

- Gamification in Medica Education PowerPoint

### Small group application exercise (sGAE)

- Cards for Candyland Gamification PowerPoint
- Cards for Candyland Gamification Answers PowerPoint

### Results and Tips for Successful Implementation

In October during a career exploration session, this gamification activity was piloted with a group of 15 learners spanning multiple levels, including second-year medical students, residents of all years (PGY-1 through PGY-4), fellows, and attendings. The didactic part of the session involving the introductory PowerPoint presentation was conducted indoors. The remainder of the session was conducted outdoors in a space large enough to allow teams to move freely along the chalk pathway. Learners were divided into small teams of 3–4 participants, intentionally composed of mixed training levels to promote camaraderie, collaboration, and incorporation of differing learner strengths and perspectives. Teams then engaged actively in answering prompts, discussing strategies, and collaborating throughout gameplay. Although formal quantitative assessment was not performed during this pilot implementation, multiple indicators suggested meaningful educational impact. Learners demonstrated active participation, collaborative problem-solving, and thoughtful application of gamification concepts during gameplay and debriefing discussions. Informal feedback indicated that participants perceived increased confidence in their understanding of gamification principles and greater readiness to incorporate gamified strategies into their own educational practice. Notably, several participants subsequently reported implementing gamified educational techniques in teaching sessions following the activity, suggesting early translational impact beyond the immediate session.

Successful implementation relied on careful preparation and consideration of logistical factors, including providing sufficient space for teams, planning a points system and rules for situations where multiple teams land on the same space, and preparing prompts in advance to ensure visibility and durability. This was an iterative process that required refinement during the initial implementation. For example, one prompt card initially awarded a disproportionately high number of points, which created a large score differential early in gameplay and appeared to reduce motivation among trailing teams. The scoring structure was subsequently modified to promote more balanced competition and sustained engagement. Additionally, when multiple teams landed on the same board space, facilitators directed teams to select previously unused prompts from completed sections of the board in order to maximize exposure to unique educational content. Minor modifications for future iterations and for publication included solidifying the points system to prevent disproportionate scores, increasing spacing between prompts to reduce crowding, and clarifying scoring instructions. These adjustments are expected to further enhance engagement while preserving the collaborative and interactive learning environment that contributed to the session’s success. Future implementations may benefit from incorporation of formal pre- and post-session assessments, learner confidence measures, or longitudinal follow-up evaluating subsequent application of gamification strategies in educational practice. If limited on physical space to play the Candy Land-based activity, future modifications can also be aimed at creating a virtual game board.

### Pearls

This session highlights the principles and practical application of gamification in medical education. Learners should understand the core concepts of gamification, including the use of game mechanics and dynamics to enhance engagement, motivation, and collaborative learning. Participants gain experience in applying gamification strategies through an interactive, team-based activity, reinforcing how games can be used to facilitate active learning, discussion, and reflection. Learners should also recognize the benefits and potential challenges of implementing gamified approaches, including logistical considerations, team dynamics, and scoring systems. Importantly, the session demonstrates how gamification can create a safe and inclusive learning environment, encouraging participation from learners across all levels of experience. By the end of the session, learners are expected to be able to conceptualize, plan, and adapt gamified activities for their own educational settings, appreciating both the theoretical foundations and practical implementation strategies necessary for success.

Ultimately, successful gamification requires intentional alignment between educational objectives and game mechanics. Educators should ensure that engagement strategies such as competition, movement, rewards, and team-based play support rather than overshadow the intended learning content. Simple rules, rapid feedback, and clearly defined educational goals can help maintain focus on knowledge acquisition while preserving learner motivation and participation. While this project was primarily designed as an educational innovation and implementation resource rather than a formal outcomes study, the observed learner engagement, reflective discussion, and subsequent application of gamification strategies suggest potential educational value warranting further study.

## Supplementary Information







## Figures and Tables

**Figure:**
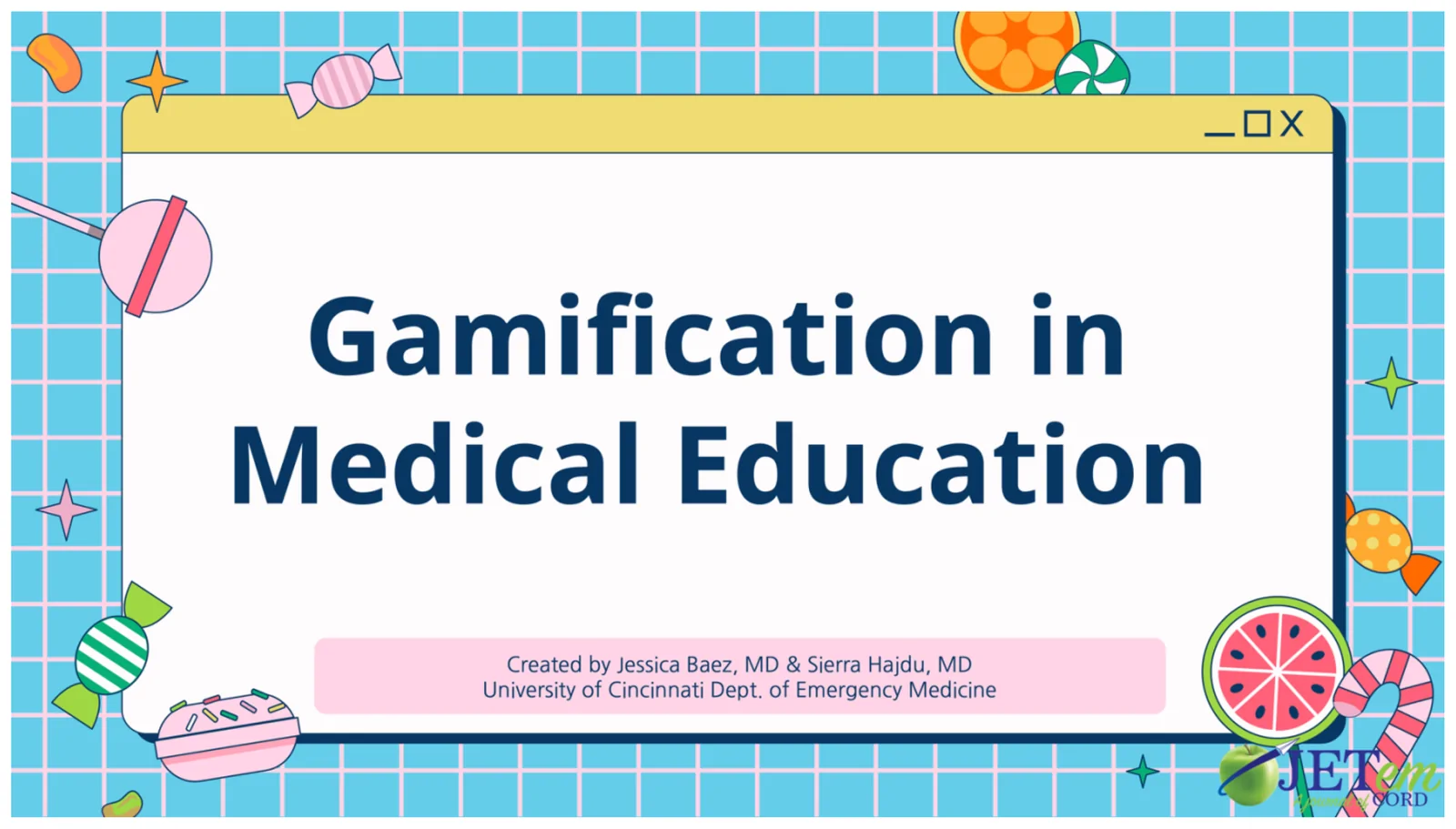
Please see associated PowerPoint file

**Figure:**
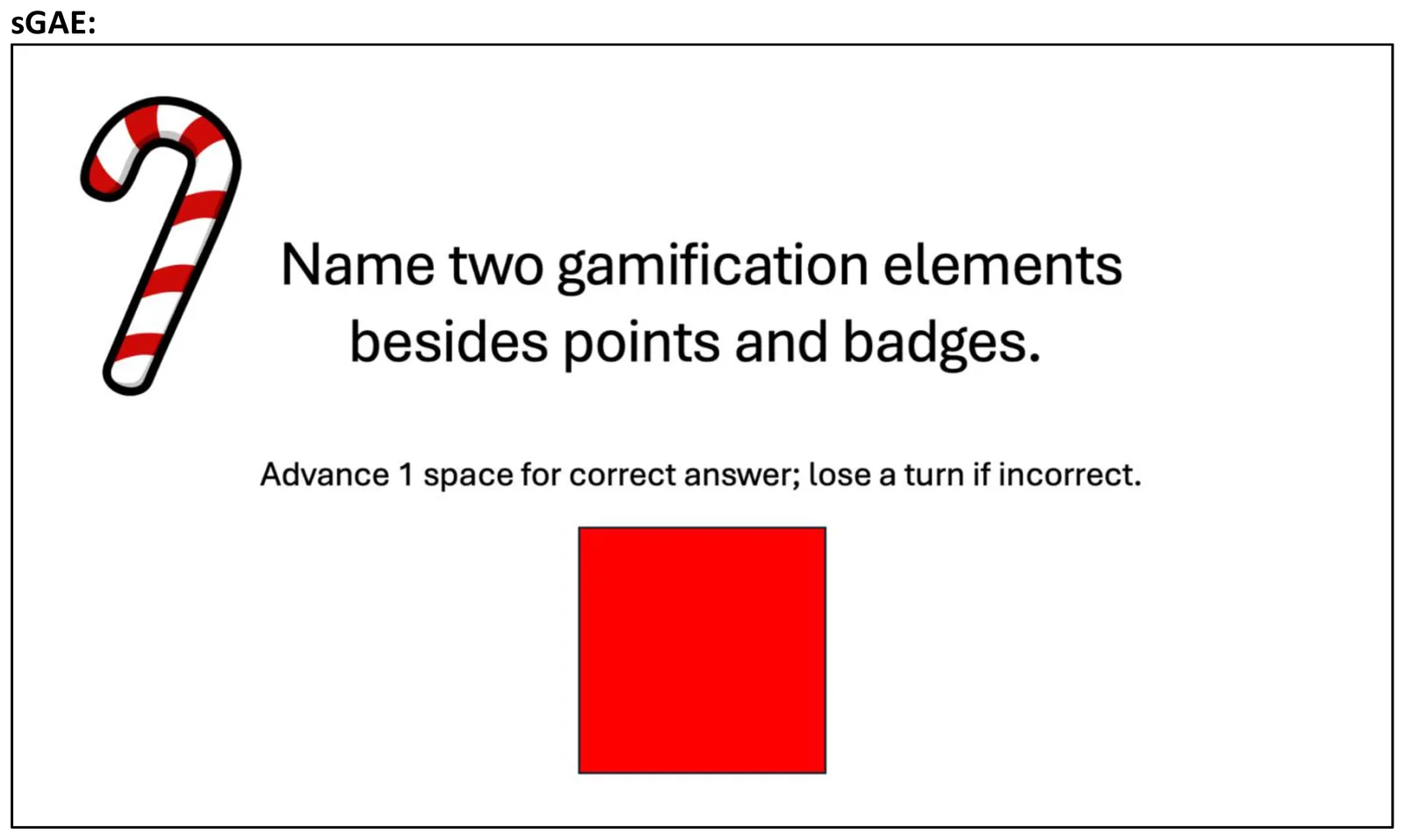
Please see associated Cards for Candyland Gamification PowerPoint file

**Figure:**
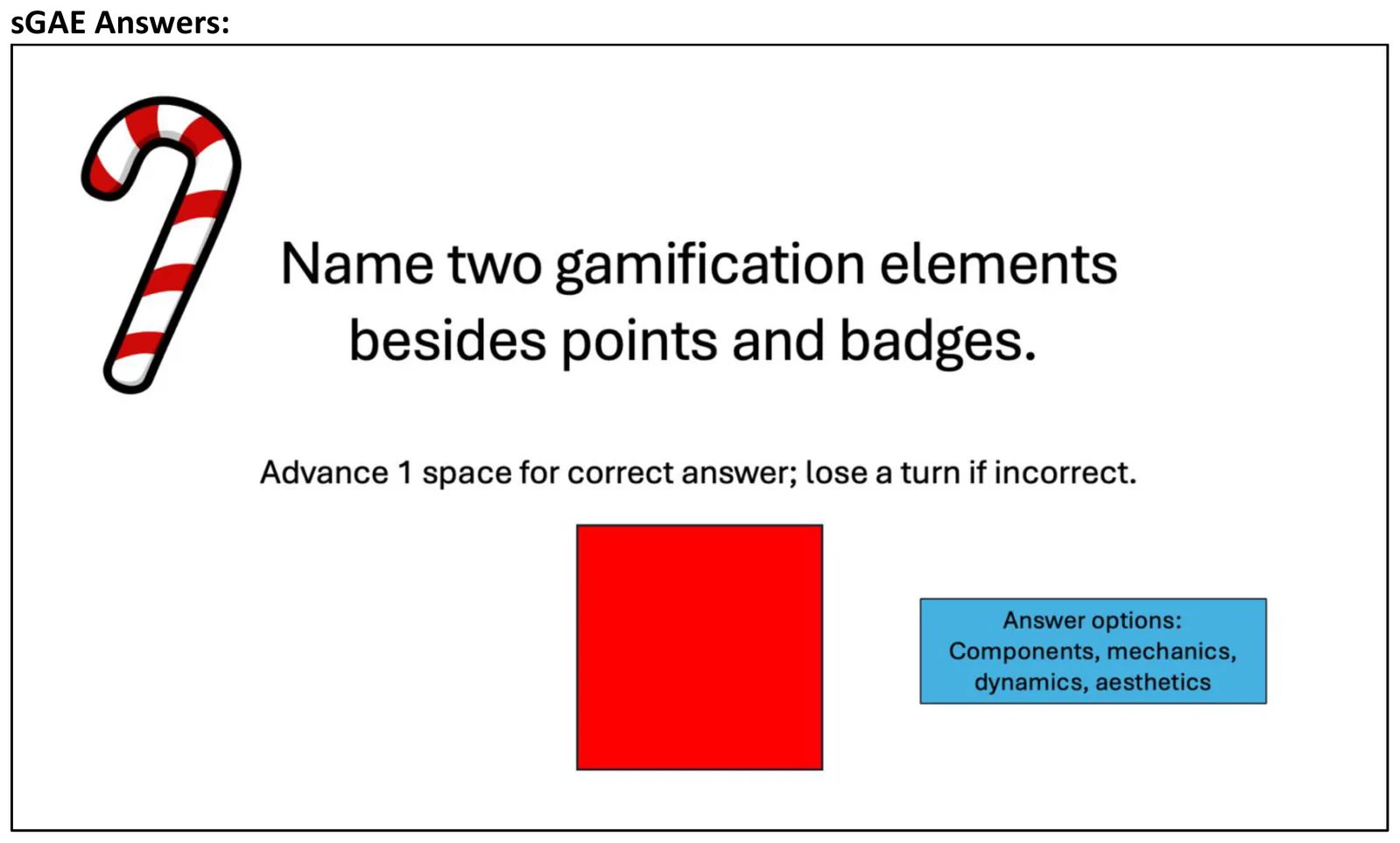
Please see associated Cards for Candyland Gamification Answers PowerPoint file

## SMALL GROUPS LEARNING MATERIALS

### Small Group Application Exercise (sGAE)

**sGAE:**

**Materials Needed:**

- PowerPoint slides (provided) containing the introductory didactic presentation, game prompts, and questions
- Colored construction paper (variety of colors)
- Scissors
- Glue sticks or tape
- Large open floor space (classroom, hallway, or conference area)
- Optional: Colored chalk or painter’s tape to draw the game pathway
- One large inflatable die (available from Amazon or similar retailers)
- Optional: Candy Land–themed decorations (eg, colored paper “tiles,” lollipops, or themed signage)

**Set-Up Instructions:**

1. **Prepare the Game Prompts:**
  ○ Print the provided PowerPoint slides on standard letter-sized paper.
  ○ Cut out each slide or prompt as needed.
  ○ Mount each printed prompt on colored construction paper for durability and visual appeal.
  ○ Arrange prompts into a desired sequence before layout.
2. **Create the Game Pathway:**
  ○ Identify a large open area where teams can move freely.
  ○ Using chalk or tape, draw a winding path on the floor with enough space between stops for players to stand or sit.
  ○ Affix each colored prompt along the path in sequential order, ensuring clear progression from “Start” to “Finish.”
3. **Prepare the Play Area:**
  ○ Designate a “Start” and “Finish” point on the path.
  ○ Place the inflatable die near the starting area.
  ○ If desired, add Candy Land–themed decorations to enhance engagement and visual appeal and contribute to the overall game aesthetic.
4. **Station Set-Up (Optional Variation):**
  ○ If running multiple groups simultaneously, divide the path into stations of 5–6 prompts each.
  ○ Assign facilitators or station leads to guide discussion and verify answers.
  ○ Each team can rotate through stations, accumulating points as they progress.
5. **Game Play Overview:** The session was designed to occur over approximately 1–2 hours, including a 15-minute introductory didactic session followed by 60 minutes of team-based gameplay and a 15-minute facilitated debriefing discussion.
  ○ Divide participants into small teams of 3–4 learners.
  ○ Teams take turns rolling the die and advancing along the path.
  ○ When landing on a prompt, the team reads the question aloud and discusses their response.
  ○ Incorrect answers are handled based on the specific prompt and intended educational objective. In some cases, teams are simply unable to advance further until the next turn, while other prompts incorporate playful consequences such as moving backward on the pathway or losing an opportunity for bonus points.
  ○ Correct answers earn allotted points or allow an additional roll as specified by each individual prompt.
  ○ The team that reaches the final prompt/finish line first receives bonus points.
  ○ The team with the highest score receives recognition at session end.

**sGAE Answers:**

**Brief wrap up (optional):** We recommend concluding the session with a facilitated group discussion designed to promote reflection and consolidate learning. The facilitator may invite participants to share their impressions of the activity, discussing what aspects of gamification were most engaging or effective and what challenges they might anticipate in implementing similar strategies in their own teaching. Participants can be asked reflective questions such as, “What did you learn about how to design or facilitate a gamified session effectively?” and “Do you feel confident that you could incorporate gamification into your own educational setting?” This debrief may allow learners to articulate key takeaways, connect the experience to their prior teaching practice, and identify opportunities for future application. By ending with guided reflection, the session can reinforce experiential learning principles and ensure participants leave with a clear sense of how to translate gamification concepts into practice.
